# Teaching basic lung isolation skills on human anatomy simulator: attainment and retention of lung isolation skills

**DOI:** 10.1186/s12871-015-0169-7

**Published:** 2016-01-20

**Authors:** Rana K. Latif, Edgar M. VanHorne, Sunitha Kanchi Kandadai, Alexander F. Bautista, Aurel Neamtu, Anupama Wadhwa, Mary B. Carter, Craig H. Ziegler, Mohammed Faisal Memon, Ozan Akça

**Affiliations:** 1 Department of Anesthesiology & Perioperative Medicine, University of Louisville, 530 South Jackson St, Louisville, KY USA; 2 University of Oklahoma, OK, USA; 3 Paris Simulation Center, Office of Medical Education, School of Medicine, University of Louisville, KY, USA; 4 Naval Medical Center, Portsmouth, VA USA; 5 Outcomes Research Consortium, Clevland, OH USA

**Keywords:** Teaching, Lung isolation skills, Comparative effectiveness, Simulation-based, Video-didactic

## Abstract

**Background:**

Lung isolation skills, such as correct insertion of double lumen endobronchial tube and bronchial blocker, are essential in anesthesia training; however, how to teach novices these skills is underexplored. Our aims were to determine (1) if novices can be trained to a basic proficiency level of lung isolation skills, (2) whether video-didactic and simulation-based trainings are comparable in teaching lung isolation basic skills, and (3) whether novice learners’ lung isolation skills decay over time without practice.

**Methods:**

First, five board certified anesthesiologist with experience of more than 100 successful lung isolations were tested on Human Airway Anatomy Simulator (HAAS) to establish Expert proficiency skill level. Thirty senior medical students, who were naive to bronchoscopy and lung isolation techniques (Novice) were randomized to video-didactic and simulation-based trainings to learn lung isolation skills. Before and after training, Novices’ performances were scored for correct placement using *pass/fail scoring* and a 5-point Global Rating Scale (GRS)*;* and *time* of insertion was recorded. Fourteen novices were retested 2 months later to assess skill decay.

**Results:**

Experts’ and novices’ double lumen endobronchial tube and bronchial blocker passing rates showed similar success rates after training (*P* >0.99). There were no differences between the video-didactic and simulation-based methods. Novices’ time of insertion decayed within 2 months without practice.

**Conclusion:**

Novices could be trained to basic skill proficiency level of lung isolation. Video-didactic and simulation-based methods we utilized were found equally successful in training novices for lung isolation skills. Acquired skills partially decayed without practice.

**Electronic supplementary material:**

The online version of this article (doi:10.1186/s12871-015-0169-7) contains supplementary material, which is available to authorized users.

## Background

Lung isolation techniques, which require special skills and experience, are used to facilitate surgical access in patients undergoing thoracic, esophageal, vascular, and non-thoracic surgical procedures [1]. There are two basic devices used to achieve lung isolation: the double lumen endobronchial tube and the bronchial blocker [2]. Traditionally, trainees acquire lung isolation skills on these devices while intubating patients under the guidance of an experienced anesthesiologist; but reportedly anesthesiology faculty and senior anesthesia residents with limited thoracic experience have a high rate of malposition or failed attempts and longer placement time [3, 4]. This method of training may result in major complications including death, and these complications may occur at rates as high as of 0.5–2 % [5–7]. Lung isolation is clearly an advanced skill, yet, how much training a novice learner requires to become proficient in this skill is unknown.

Traditionally, in educational research, either one method of teaching is compared with no teaching [8] or with less robust teaching [9]. However, since simulation [10–14] and video-based trainings [15–18] have become established as powerful teaching tools, we now have a stronger standing in doing comparative effectiveness studies on instruction techniques [19].

Repetitive practice is required to perform a rarely used procedure upon short notice [20], and such skills decay if not actively maintained with repetitive practice [21]. The deficiency or decay of lung isolation skill among anesthesiologists is costly as it can result in delayed or cancelled surgeries [22], prolonged operating room time [23], and airway complications [5–7].

Therefore, we planned a study to find out whether we can train novice learners to basic level of proficiency in lung isolation skills without risking our patients. Our detailed study aims were whether 1) novices can be trained to basic proficiency level of lung isolation skills before they apply their skills in patients; 2) video-based training is comparable to simulation-based training in teaching basic lung isolation skills; 3) lung isolation skills decay in a short time (2 months) with no practice.

## Methods

The study was submitted to the Human Studies Committee and Human Subjects Protection Program Office of the Institutional Review Board (IRB), University of Louisville, Kentucky, USA. It was determined by the IRB that the study is ‘exempted’ according to title 45 CFR (Code of Federal Regulations) part 46.101(b). The need for informed consent and submission of progress reports for continuous reviews were judged unnecessary by the IRB. This study was conducted in the Paris Satellite Simulation Center at the University of Louisville Hospital, Louisville, Kentucky, USA.

### Subjects

#### Expert group (*n* = 5)

Board certified anesthesiology faculty, who had been routinely practicing thoracic anesthesia and also teach lung isolation techniques to residents on a regular basis, were recruited to form the Expert group. Each expert had performed more than 100 successful lung isolations with both the double lumen endobronchial tube and the bronchial blocker techniques.

#### Experienced group (*n* = 9)

Senior anesthesia residents who were within 6 months of completing their anesthesia training and who had previously performed more than 20 successful lung isolations were recruited as the Experienced group. Twenty cases are the number required by Accreditation Council for Graduate Medical Education (ACGME) in anesthesiology during residency [24].

#### Novice group (*n* = 30)

Senior medical students, who were previously trained as members of Anesthesia Interest Group in endotracheal intubation with direct laryngoscopy on simulator but with no previous experience in bronchoscopy, fiberoptic- bronchoscopy assisted intubation or lung isolation, were recruited as the Novice group. The numbers of attempts required by the novices to successfully intubate the simulator with direct laryngoscopy before enrolling in this study were not recorded.

### Simulation set up

**Fig. 1:**
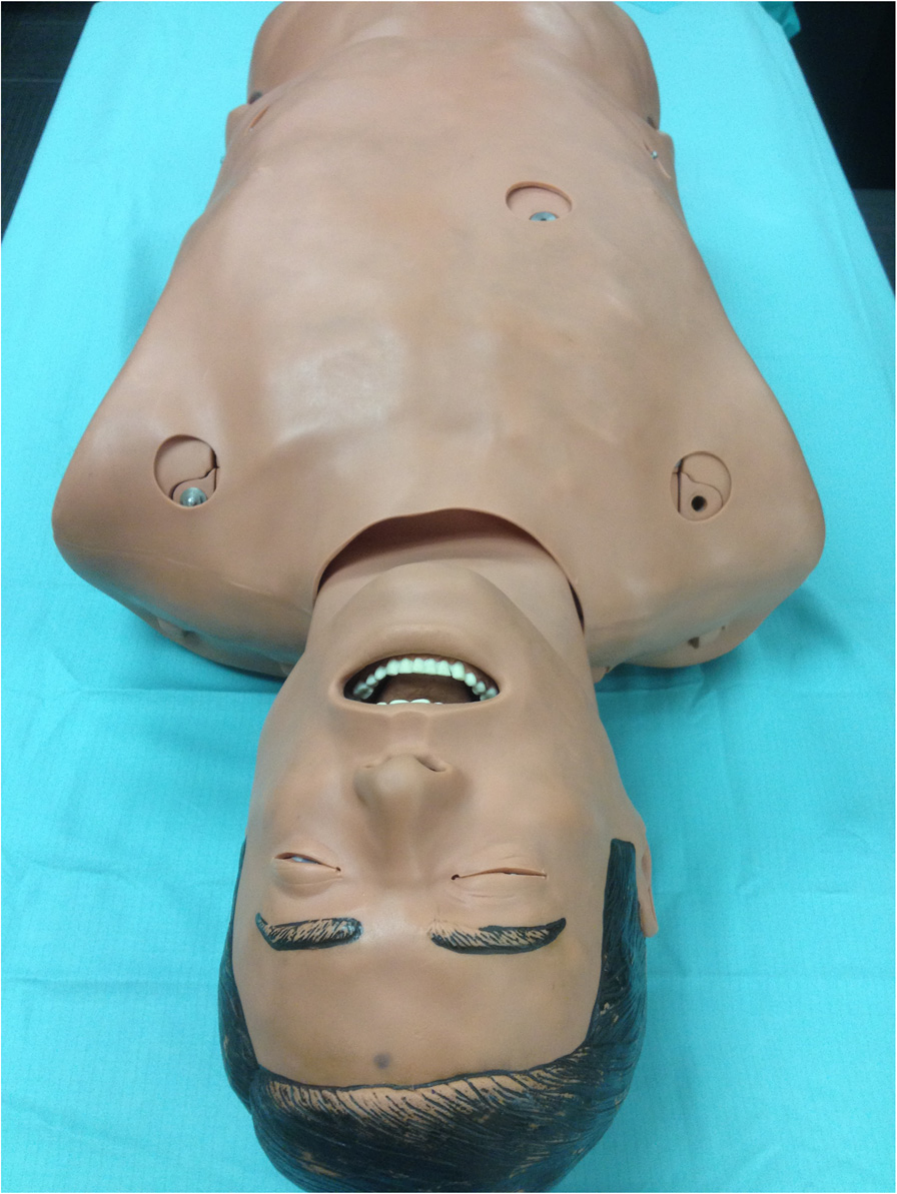
The Human Airway Anatomy Simulator (HAAS)

In this research, we utilized The Human Airway Anatomy Simulator (Medical Plastic Laboratory, Gatesville, TX) with simulated carina bifurcating into a right and left main-stem bronchus only (Fig. 1)

.

### Protocol

Expert subjects performances were assessed to establish basic proficiency in lung isolation skills. Experienced group were tested similarly.

#### Establishment of proficiency

On the HAAS, Expert subjects performed bronchial blocker (Arndt Blocker, Cook Medical, Bloomington, IN) and double lumen endobronchial tube (Mallinckrodt, 35Fr, Covidien, Ireland) insertion.

For the bronchial blocker data collection, a stopwatch was started as soon as the tip of the bronchoscope entered the endotracheal tube and was stopped when the subject declared that the bronchial blocker was correctly placed. Four photographic images were recorded to document the position of the bronchial blocker and details are in Additional file 1.

For the double lumen endobronchial tube device insertion, Expert subjects were instructed to perform oral intubations with direct laryngoscopy and blind advancement of the double lumen endobronchial tube into left main bronchus, followed by repositioning using the FOB. A stopwatch was started as soon as the tip of the double lumen endobronchial tube passed the lips and was stopped when the subject declared that the tube was correctly placed and positioning was photo-documented (Additional file 1). Experts were not aware of the contents of the checklist and the Global Rating Scale, which was subsequently used to rate their performance of correct lung isolation skill testing.

The mean *time* in seconds Experts took to complete lung isolation with each device was calculated and considered the benchmark time for proficiency level of performance for the remainder of this study.

#### Training and assessment of novices

##### Baseline assessment

Initially, Novices received a 15-min didactic lecture included familiarity with structure and functions of fiberoptic scope. For bronchial blocker, how to use the FOB to place it into left main bronchus. For the double lumen endobronchial tube, how to turn the device 90° to the left after passing the vocal cords, and position it in the left main bronchus. At this session, Novices were not given the opportunity for “hands-on” practice. Novices were shown what constituted *correct placement* and *incorrect placement* of the bronchial blocker and double lumen endobronchial tube devices for left main stem intubation based on previously used criteria (Table 1) [3, 25]. Subsequently, all Novices performed one bronchial blocker and one double lumen endobronchial tube placement on the HAAS to establish their baseline skills level. Time required to complete each procedure and photographic documentation of placement of each device were recorded (Additional file 1).

**Table 1:** Criteria used for Novices to assess correct placement and incorrect placement of the bronchial blocker and double lumen endobronchial tube

*Criteria to assess malposition:*
• More than 50 % of bronchial cuff herniated into carina (too far out)
• Bronchial cuff edge not visible in the entrance of main-stem bronchus such that it would occlude secondary bronchus (too far in)
• Double- lumen endotracheal tube or Bronchial Bronchus in the opposite bronchus (Right instead of Left)
• Unable to distinguish tracheal/bronchial anatomy
*Criteria to assess correct placement:*
• The bronchial cuff edge just visible in left main bronchus

Scoring criteria [3, 25] used with permission from the author (Campos JH)

##### Training methods

Subsequently, Novices were randomized into two groups, the Video-didactic group and the Simulation-based group, using a computer-generated sequence by simple randomization with a 1:1 allocation ratio.

i. Simulation-based training and post-training testing
  Subjects who were randomized to the Simulation-based group performed multiple bronchial blocker and double lumen endobronchial tube placements on the HAAS under direct supervision by a thoracic anesthesiologist (who was a different professional than the Expert group performers). Training ceased once the subject was able to achieve previously set proficiency benchmark of insertion *time* and correct placement [3, 25]. Finally, the Simulation-based group underwent Post-Training testing on bronchial blocker and double lumen endobronchial tube insertion.
ii. Video-didactic training and post-training testing
  Subjects randomized to the Video-didactic group watched previously prepared videos about double lumen endobronchial tube insertion and bronchial blocker placement (Arndt Endobronchial Blocker, Cook Medical, Bloomington, IN, C-MCD-AEBSM1205) followed by post training testing. The video clips explained all steps involved with each technique and what constituted *correct* and *incorrect placement* of each device. The Novices watched the two video clips only once before undergoing testing. At this session, Novices were not given the opportunity for “hands-on” practice.
iii. Skill decay testing
  The goal of this stage of the study was to determine whether any skill decay had occurred during the 2 months without practice. We selected 2 months of no use for skill decay as residents usually spend 1 to 2 months in each clinical rotation before moving to the next.

### Blinded photograph rating

The recorded pictures were evaluated with previously used criteria [3, 25] with permission (Table 1).

### Data analysis

Group differences between the Expert, Experienced, Simulation-based and Video-didactic for the final sum correct placement Global Rating Scale (GRS) scores were compared by the Kruskal Wallis and Mann-Whitney U test; the change in novices scores across time were assessed by Wilcoxon Signed Rank test. The inter-rater reliabilities between the two expert raters were assessed by weighted Kappa for GRS scores and the Kappa statistics for pass/fail. The McNemar test was used to compare the novices’ passing rates between the pre-training and post-training and 2-month follow-up time; the proportion difference and 95 % confidence intervals for differences of two binomial proportions were performed as well.

The assumptions of parametric statistics were checked for the total time (seconds) required to complete the procedure data. Because these data were non-normally distributed, square root transformations were applied to the data leading to near normal distributions. One-way analysis of variance was employed to compare the differences between the Expert, Experienced, Simulation-based, and Video-didactic Novice groups. Paired samples t-tests were used to compare the novices’ pre-training, post-training and 2-month follow-up times.

Data were analyzed using SPSS (version 20.0). Owing to multiple, paired samples for t-tests performed to assess novices’ times for pre-training, post-training and 2-month follow-up, a Bonferroni correction was used and statistical significance was set at *P* <0.017. For all other analyses, statistical significance was set by convention at *P* <0.05.

## Results

The total duration of time for initial training of each Novice was approximately 90 min, including lecture, orientation with the equipment, watching the training video or training with simulator, and final performance assessment.

**Fig. 2:**
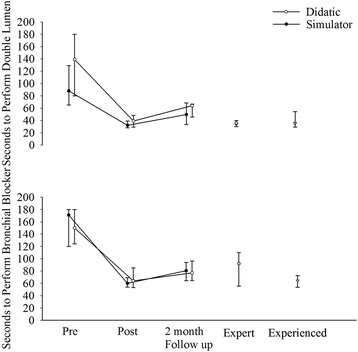
Comparison of lung isolation time (seconds) in the Human Airway Anatomy Simulator in Experts, Experienced and Novice Groups (Pre and post Simulation-based and Video-didactic training groups). Novice times at a 2-month follow-up evaluation are also presented. Data presented as medians with interquartile range. Statistical significance: Experts and Experienced vs. Novice Pre-Training groups (Simulation and Video; Double lumen and Bronchial Blocker) - *P* <0.001; Novice Pre-Training vs. Novice Post-Training (Simulation and Video; Double lumen and Bronchial Blocker) - *P* <0.001. Novice Post-Training vs. 2 month Follow up (Double lumen and Bronchial Blocker) - *P* <0.01. Novice Pre-Training vs. 2 month Follow up (Double lumen and Bronchial Blocker) - *P* <0.01

There were no differences among the Expert group and Novice groups after the training in either time or correct placement criteria (Fig. 2 and Table 2). Similarly, there were no differences between the Simulation-based and Video-didactic groups after the training in any of the lung isolation skills’ assessments (Fig. 2 and Table 2). The 15 novices in Simulation-based group required 3.3 ± 2.2 double lumen endobronchial tube attempts and 2.9 ± 1.3 bronchial blocker attempts to reach basic proficient level in simulator.

**Table 2:** Comparison of lung isolation skill in the Human Airway Anatomy Simulator in Experts, Experienced and Novice Groups (Simulation-based vs. Video-didactic)

	Experts (*n* = 5)	Experienced (*n* = 9)	Novices				
			*Pre-Training (n = 30)*		*Post-Training (n = 30)*		
			Simulation (*n* = 15)	Video-Didatic (*n* = 15)	Simulation (*n* = 15)	Video-Didatic (*n* = 15)	Both Novice groups (*n* = 30)
Pass/Total (%)^a^							
*DLT*	5/5 (100 %)	8/9 (89 %)	4/15 ^c^ (27 %)	6/15 ^c^ (40 %)	14/15 ^d^ (93 %)	14/15 ^d^ (93 %)	28/30 (93 %)
*Bronchial Blocker*	5/5 (100 %)	9/9 (100 %)	1/15 ^c^ (7 %)	4/15 ^c^ (27 %)	13/15 ^d^ (87 %)	15/15 ^d^ (100 %)	28/30 (93 %)
GRS score^b^							
*DLT*	4.7 ± 0.4	4.4 ± 0.6	2.2 ± 1.2 ^c^	2.5 ± 1.2^c^	4.3 ± 0.6^d^	4.1 ± 0.6^d^	4.2 ± 0.7
*Bronchial Blocker*	4.8 ± 0.3	4.7 ± 0.3	1.9 ± 1.1^c^	2.0 ± 1.0^c^	4.1 ± 0.8^d,e^	4.6 ± 0.5^d^	4.3 ± 0.7

Data are expressed as Mean ± SD. DLT = Double Lumen Tube; ^a^ Pass = Number of subjects in the group who passed; Total = Number of subjects in group, % = percentage of subjects who passed in that group. ^b^GRS = Global Rating Scale. Scored as “Very poor” to “Excellent” (Maximum Score is 5). The average of the two evaluators is the final sum checklist score. ^c^ *P* <0.001 Experts/Experienced vs. Novices Pre-Training (Simulation and Video). ^d^ *P* <0.001 Novice Pre-Training vs. Novices Post-Training (Simulation and Video). ^e^ *P* = 0.039 Expert vs. Post-Training Simulation Group

Of the original 30 Novices, 14 returned within 2 months for repeat testing. Compared with their post-testing performance, there was significant *skill decay* after 2 months of no practice as shown by an increase in time to correct placement. This skill data is presented as medians with interquartile range (Fig. 2).

However, they also showed a significant *retention of lung isolation skills* in that they tested significantly better at the 2-month evaluation as compared with their baseline pre-training scores in (1*) time to placement of both devices* (Fig. 2); (2) the 5-point GRS scores for double lumen endobronchial tube placement (pre-training 2.4 ± 1.2 vs. 2-month 3.8 ± 0.9; *P* <0.01); (3) GRS scores for bronchial blocker placement (pre-training 1.9 ± 1.1 vs. 2-month 4.3 ± 0.6, *P* <0.01); (4) “pass” percentage for double lumen endobronchial tube placement (pre-training 29 % [4/14] vs. 2-month 93 % [13/14]; difference of 64 % [30–87 %], *P* <0.01); and (5) “pass” percentage for bronchial blocker placement (pre-training 21 % [3/14] vs. post training 100 % [14/14]; difference of 79 % [48–95 %], *P* <0.01).

Inter-rater reliability, as measured by Kappa and weighted Kappa, on the double lumen and bronchial blocker pass/fail and rating scales ranged from fair to good. For the pass/fail data, Kappa varied from 0.38 to 0.61, *P* <0.01. For the rating scales, weighted Kappa spanned from 0.34 to 0.57, *P* <0.05.

A *post hoc* or retrospective power calculation was performed on several outcome measures to assess if the absence of statistical difference between groups resulted from a Type II error. For the Global Rating Scale (GRS) for double lumen endobronchial tube outcome, group sample sizes of 5 (expert) and 15 (post-training simulation novices) would achieve 90 % power to detect a 20 % difference between the null hypothesis (that both group means are 4.7) and the alternative hypothesis (that the mean of the simulation group is 3.8), using a two-sided Mann-Whitney U test and a significance level of 0.05. Ninety-three percent power was detected for the Global Rating Scale for bronchial blocker outcome based on a 20 % mean difference between the expert (mean = 4.8) and post training simulation groups (mean = 3.8). Regarding the double lumen endobronchial tube time outcome, the group sample sizes of 5 (expert) and 15 (post-training simulation novices) would achieve 85 % power to detect a 40 % difference between the null hypothesis (that both group means are 35 s) and the alternative hypothesis (that the post-training simulation group mean is 48 s). No retrospective power calculation was performed on the bronchial blocker time outcome as the post-training simulation group performed faster than the expert group (post-training simulation mean time of 62 s vs. experts mean time of 85 s). Similarly, a power analysis was not performed on the double lumen endobronchial tube and bronchial blocker percentages passing as the post-training passing rates were much greater than those found in Campos’ study [3].

## Discussion

In this study, we trained novices with two different training techniques. Our data indicated that Video-based didactic training was as effective as Simulation-based training in teaching basic lung-isolation skills. A part of the skills decayed within a 2-month period of no practice.

To advance the science of teaching, Cook et al. suggested applying the principles of comparative effectiveness of clinical research to educational research; comparing one teaching method to another [19]. In this study, we developed performance measurement instruments, established a proficient competency level, trained faculty to rate the subjects’ performance, and also compared two active interventions in training novices to basic proficient competency level. Although there are many excellent tools available in the literature to assist in lung isolation learning [26], there is no single work about how to train novices to competency level for basic skill set, and when and how to retain them. We have provided a systems-based guidance tool on how to introduce an important technical skill to novice learners, how to test them, and when to retrain for decay in skills.

*Skill decay* refers to loss of acquired skill, knowledge, or training after a period of non-use [21]. The longer the period of non-practice or non-use, the greater the decay will be [21, 27]. In our study, there was a statistically significant decay of insertion speed of 15 s, but that may have little clinical significance. More than 50 % novices could not be available for the skill decay retesting at 2-months for the allowed time period. This may have affected our presented results positively or negatively.

Our skill decay over time data suggests that non-thoracic anesthesiologists may require on-going re-enforcement of their lung isolation skills by performing certain numbers of successful procedures each year. We used 20 successful lung isolation procedures for the Experienced group as this is the minimum number required by Accreditation Council for Graduate Medical Education (ACGME) in anesthesiology during three years of residency [24]. We selected more than 100 successful lung isolation for the expert group because minimum number of successful procedure is the only way to certify proficiency at this moment.

In our study, we used senior year medical students as novices instead of junior anesthesiology residents. One aim of the study was to determine *skill decay* after fixed period of non-practice or non-use. Because our hospital is a Level-1 trauma center, our residents may perform lung isolation as a part of their normal clinical work. Therefore, we could not pick our novice learners from our residents. This potentially would have invalidated the skill decay data.

There are various limitations to our results. First, we acknowledge that the HAAS is made of plastic and there cannot be any difficult airway or harm such as migration of lung isolation device, hypoxia, tension pneumothorax, pneumomediastinum, pneumoperitoneum and tracheobronchial rupture due to poor lung isolation technique [5–7]. However, if we had performed this study in patients, the assessment would have faced many other limitations: a) the time between completion of training to testing of lung isolation skill would be different in each trainee, which would make the comparison difficult; b) testing conditions could not have been standardized because each patient’s anatomy is different; [25] and c) the assessment of dexterity decay after fixed lapse of time in double lumen endobronchial tube and bronchial blocker would have been impossible on patients for similar reasons mentioned above (please see “a”). Therefore, the value of training and testing novices on human anatomy simulators is both obvious and inevitable in teaching the basic skill set.

The Human Airway Anatomy Simulator (HAAS) has a carina, which bifurcates into a right and left main-stem bronchus only. In patients with normal anatomy, the malposition lung isolation devices can be identified and corrected by fiberoptic bronchoscope assisted visualization of right upper lobe bronchus with its apical, anterior and posterior segmental bronchus, not present in HAAS. Similarly, in patients with congenital and acquired anomalies of the tracheobronchial tree, the differentiation of tracheal anatomy and lung isolation can be very challenging [28, 29]. In summary, the unfamiliarity with the use of fiberoptic scope and anatomy of the tracheobronchial tree can increase placement time with decrease success rate in patients [3, 4].

## Conclusion

In conclusion, in this comparative effectiveness educational study, we concluded that novice learners can be trained to basic skill level of lung isolation proficiency in the simulator setting. We also found out that video-based didactic learning was as effective as our simulation-based training. These newly learned lung isolation skills begin to decay within 2 months after initial training without practice. The skill decay findings may enforce the concept of advance procedural skills -such as elective lung isolation- to be only practiced by sub-specialists who practice these skills routinely. Alternatively, one may conclude that anesthesiologists who do not practice thoracic anesthesia and lung isolation skills routinely may require reinforcement/retraining of advanced skills in some intervals. Future research should focus on translating the lung isolation skills learned on simulators to real patients, who have non-standard and possibly difficult anatomy, as well as different body habitus.

## Additional file

Additional file 1:
**Appendix 1.** Photographs and photograph assessment tool. (DOCX 18 kb)

## References

[1] Campos JH (2001). Lung isolation techniques. Anesthesiol Clin North America.

[2] Campos JH (2002). Current techniques for perioperative lung isolation in adults. Anesthesiology.

[3] Campos JH, Hallam EA, Van Natta T, Kernstine KH (2006). Devices for lung isolation used by anesthesiologists with limited thoracic experience: comparison of double-lumen endotracheal tube, Univent torque control blocker, and Arndt wire-guided endobronchial blocker. Anesthesiology.

[4] Campos JH, Kernstine KH (2003). A comparison of a left-sided Broncho-Cath with the torque control blocker univent and the wire-guided blocker. Anesth Analg.

[5] Sivalingam P, Tio R (1999). Tension pneumothorax, pneumomediastinum, pneumoperitoneum, and subcutaneous emphysema in a 15-year-old Chinese girl after a double-lumen tube intubation and one-lung ventilation. J Cardiothorac Vasc Anesth.

[6] Fitzmaurice BG, Brodsky JB (1999). Airway rupture from double-lumen tubes. J Cardiothorac Vasc Anesth.

[7] Sucato DJ, Girgis M (2002). Bilateral pneumothoraces, pneumomediastinum, pneumoperitoneum, pneumoretroperitoneum, and subcutaneous emphysema following intubation with a double-lumen endotracheal tube for thoracoscopic anterior spinal release and fusion in a patient with idiopathic scoliosis. J Spinal Disord Tech.

[8] Latif R, Chhabra N, Ziegler C, Turan A, Carter MB (2010). Teaching the surgical airway using fresh cadavers and confirming placement nonsurgically. J Clin Anesth.

[9] Latif RK, Bautista AF, Memon SB, Smith EA, Wang C, Wadhwa A (2012). Teaching aseptic technique for central venous access under ultrasound guidance: a randomized trial comparing didactic training alone to didactic plus simulation-based training. Anesth Analg.

[10] Martin KM, Larsen PD, Segal R, Marsland CP (2004). Effective nonanatomical endoscopy training produces clinical airway endoscopy proficiency. Anesth Analg.

[11] Naik VN, Matsumoto ED, Houston PL, Hamstra SJ, Yeung RY, Mallon JS (2001). Fiberoptic orotracheal intubation on anesthetized patients: do manipulation skills learned on a simple model transfer into the operating room?. Anesthesiology.

[12] Barsuk JH, Cohen ER, Feinglass J, McGaghie WC, Wayne DB (2009). Use of simulation-based education to reduce catheter-related bloodstream infections. Arch Intern Med.

[13] Barsuk JH, McGaghie WC, Cohen ER, O’Leary KJ, Wayne DB (2009). Simulation-based mastery learning reduces complications during central venous catheter insertion in a medical intensive care unit. Crit Care Med.

[14] Marsland CP, Robinson BJ, Chitty CH, Guy BJ (2002). Acquisition and maintenance of endoscopic skills: developing an endoscopic dexterity training system for anesthesiologists. J Clin Anesth.

[15] Kamin C, O’Sullivan P, Deterding R, Younger M (2003). A comparison of critical thinking in groups of third-year medical students in text, video, and virtual PBL case modalities. Acad Med.

[16] Balslev T, de Grave WS, Muijtjens AM, Scherpbier AJ (2005). Comparison of text and video cases in a postgraduate problem-based learning format. Med Educ.

[17] Makary MA. The power of video recording: taking quality to the next level. JAMA. 2013;1–2. 10.1001/jama.2013.595 23546452

[18] Hu YY, Peyre SE, Arriaga AF, Osteen RT, Corso KA, Weiser TG (2012). Postgame analysis: using video-based coaching for continuous professional development. J Am Coll Surg.

[19] Cook DA (2012). If you teach them, they will learn: why medical education needs comparative effectiveness research. Adv Health Sci Educ.

[20] Issenberg SB, McGaghie WC, Petrusa ER, Lee Gordon D, Scalese RJ (2005). Features and uses of high-fidelity medical simulations that lead to effective learning: a BEME systematic review. Med Teach.

[21] Arthur Jr. WBJ, Winston; Stanush, Pamela L.; McNelly, Theresa L. Factors that influence skill decay and retention: a quantitative review and analysis. Human Performance. 1998;11(1):57–101

[22] Lau HK, Chen TH, Liou CM, Chou MC, Hung WT (2010). Retrospective analysis of surgery postponed or cancelled in the operating room. J Clin Anesth.

[23] Macario A (2010). What does one minute of operating room time cost?. J Clin Anesth.

[24] The Accreditation Council for Graduate Medical Education: ACGME Program Requirements for Graduate Medical Education in Anesthesiology. http://www.acgme.org/acgmeweb/portals/0/pfassets/programrequirements/040_anesthesiology_07012014.pdf. Accessed 30 November 2015

[25] Campos JH, Hallam EA, Ueda K (2012). Lung isolation in the morbidly obese patient: a comparison of a left-sided double-lumen tracheal tube with the Arndt(R) wire-guided blocker. Br J Anaesth.

[26] Campos JH, Hallam EA, Ueda K (2011). Training in placement of the left-sided double-lumen tube among non-thoracic anaesthesiologists: intubation model simulator versus computer-based digital video disc, a randomised controlled trial. Eur J Anaesthesiol.

[27] Ahya SN, Barsuk JH, Cohen ER, Tuazon J, McGaghie WC, Wayne DB (2012). Clinical performance and skill retention after simulation-based education for nephrology fellows. Semin Dial.

[28] Desir A, Ghaye B (2009). Congenital abnormalities of intrathoracic airways. Radiol Clin N Am.

[29] Sangster GP, Gonzalez-Beicos A, Carbo AI, Heldmann MG, Ibrahim H, Carrascosa P (2007). Blunt traumatic injuries of the lung parenchyma, pleura, thoracic wall, and intrathoracic airways: multidetector computer tomography imaging findings. Emerg Radiol.

