# Supplementary material for: Teaching basic lung isolation skills on human anatomy simulator: attainment and retention of lung isolation skills
Source: BMC Anesthesiol. 2016 Jan 20;16:7. doi: 10.1186/s12871-015-0169-7 (PMC4719687; doi:10.1186/s12871-015-0169-7)
Supplement: Additional file 1: — Appendix 1. Photographs and photograph assessment tool. (DOCX 18 kb) [file 12871_2015_169_MOESM1_ESM.docx]

**Additional file 1**

**Appendix 1**

**Photographs and photograph assessment tool**

Photographs:

For correct placement assessment of lung isolation devices, four photographic images were recorded to photo-document the position of the bronchial blocker and double lumen endobronchial tube in relation to the carina. The images were printed on a single page and assigned a random number.

*Photograph Assessment Tool*:

1. *“Pass/Fail” Instrument*: We selected a previously used scoring instrument [3, 25] (with permission) and added “pass/fail” rating. A “pass” means, no further training is required and “fail” means, further training is required. We removed one item from the instrument, “if a right main-stem bronchus intubation was planned, the blocker was distal to origin of the right upper lobe bronchus”, since right main-stem bronchus intubation was not used in this study (Table 1).
2. *Global Rating Scale (GRS):* We added a 5-point Likert-like Global Rating Scale (Table 1).

*Training the Raters:*

Two of our investigators, who are experienced trauma anesthesiologists, AN and AW, served as blinded raters in this study. They were trained in the use of Photograph Assessment Tool, what constituted a ‘Pass’ and a ‘Fail’ and on the overall quality of using the GRS.
